# H3K18la facilitates nasopharyngeal carcinoma progression through the activation of the IGF2BP1-COX2 signaling pathway

**DOI:** 10.3389/fimmu.2026.1864228

**Published:** 2026-09-08

**Authors:** Zhaomeng Guo, Dunhui Yang, Ruyu Niu, Lisi Su, Zhen Wang, Fang Ma, Peng Zhang

**Affiliations:** 1 Department of Otolaryngology, Shenzhen Longgang Otolaryngology Hospital & Shenzhen Otolaryngology Research Institute, Shenzhen, China; 2 School of Basic Medical Sciences, Changzhi Medical College, Changzhi, China

**Keywords:** COX2, H3K18la, IGF2BP1, lactate, nasopharyngeal carcinoma

## Abstract

Nasopharyngeal carcinoma (NPC) is a malignant tumor arising from the nasopharyngeal epithelial mucosa, with its development closely associated with metabolic reprogramming. Lactylation, an epigenetic modification induced by lactate, plays a regulatory role in gene transcription.The precise function and mechanisms of H3K18la in NPC malignancy progression are yet to be clarified.This study demonstrates that the glycolytic pathway is markedly activated in NPC tissues, accompanied by elevated expression of H3K18la, which positively correlates with tumour stage. Subsequent studies showed that lactic acid production, mediated by lactate dehydrogenase A (LDHA), facilitates H3K18la modification. Inhibiting LDHA specifically leads to reduced H3K18la levels, suppressed proliferation of NPC cells, and increased apoptosis.H3K18la upregulates the RNA-binding protein IGF2BP1, while inhibiting LDHA or IGF2BP1 reduces COX2 mRNA stability and expression, influencing NPC cell proliferation and apoptosis resistance. In summary, this study indicates that H3K18la modification is crucial for NPC malignancy through the activation of the IGF2BP1-COX2 signaling pathway. This research elucidates lactylation modification mechanisms in NPC, presenting novel targets for its treatment.

## Introduction

Nasopharyngeal carcinoma (NPC) is a malignant tumor arising from the nasopharynx’s epithelial lining, characterized by significant regional distribution and epidemiological differences. NPC exhibits a high incidence in East and Southeast Asia, contrasting with its lower prevalence in Western regions like Europe and the United States (1). Consequently, it is colloquially referred to as “Guangdong cancer.” The development of NPC is influenced by multiple factors, including Epstein-Barr virus (EBV) infection, genetic susceptibility, environmental factors, and immune-inflammatory responses (2). Among these, EBV infection is identified as the primary etiological agent; chronic EBV infection and the resultant gene expression alterations are believed to drive the oncogenic transformation of nasopharyngeal epithelial cells (3). The standard treatment for NPC primarily combines radiotherapy and chemotherapy, significantly improving local disease control and patient survival rates. Therefore, thoroughly examining the molecular mechanisms underlying malignant progression is crucial for improving clinical diagnosis and treatment strategies for NPC.

Lactylation, a newly discovered post-translational modification, redefines lactate’s role beyond being a glycolysis byproduct that promotes tumor progression by acidifying the tumor microenvironment. Recent studies suggest that lactate acts as a substrate for histone post-translational modification by lactylating lysine residues, which affects gene transcription (4). Histone lactylation in cancer research is linked to enhancing malignant traits such as tumor growth, migration, and apoptosis resistance in diverse cancers. For instance, in pancreatic ductal adenocarcinoma, the interplay between glycolysis and histone lactylation is a driving force behind tumorigenesis (5). In breast cancer, histone lactylation enhances the PI3K/AKT/HIF-1α signaling pathway by increasing USP39 expression, promoting malignant progression (6). In hepatocellular carcinoma, PYCR1 influences IRS1 expression through lactic acid modification, promoting cell proliferation (7). Nonetheless, the expression patterns, functions, and mechanisms of histone lactic acid modification in NPC remain inadequately understood, necessitating further investigation into its specific oncogenic mechanisms.

Histone H3 lysine 18 lactylation (H3K18la) is a newly discovered modification at the lysine 18 residue of histone H3, playing a crucial role in epigenetic regulation. H3K18la lactylation in non-small cell lung cancer increases the tumor’s ability to evade the immune system (8). Conversely, in ovarian cancer, it inhibits tumor ferroptosis and reduces sensitivity to cisplatin (9). However, the expression profile and clinicopathological significance of H3K18la in NPC remain insufficiently characterized. The Insulin-like Growth Factor 2 mRNA Binding Protein 1 (IGF2BP1) gene is associated with drug resistance and immune evasion in multiple tumors. However, its regulation by H3K18la in NPC and the specific mechanisms involved are not yet understood and require further investigation.

In this study, we employed bioinformatics and immunohistochemistry to characterize the expression profile of H3K18la in NPC and its association with clinical staging. Additionally, we elucidated the regulatory influence of LDHA-mediated lactate production on H3K18la modification. We conducted an in-depth investigation into the molecular mechanism by which H3K18la modulates the malignant phenotype of NPC via the activation of the IGF2BP1-COX2 axis, suggesting novel theoretical insights and identify potential targets for the metabolic and precision-targeted treatment of NPC.

## Materials and methods

### Data sources and enrichment analysis

The NPC datasets GSE12452 and GSE64634 were sourced from the GEO public database (https://www.ncbi.nlm.nih.gov/geo/) (10). Normalization was performed using the “normalizeBetweenArrays” function from the “limma” R package. Differentially expressed genes were identified with criteria of |logFC| > 1 and P < 0.05 (11).The ‘clusterProfiler’ R package (12)was used to conduct GSEA analysis on the differentially expressed genes.

### Cell culture

The BeNa Culture Collection in Jiangsu, China, provided the human nasopharyngeal carcinoma cell lines C666–1 and 5-8F. Cells were cultured in DMEM or RPMI 1640 medium supplemented with 10% fetal bovine serum, 100 U/mL penicillin, and 100 μg/mL streptomycin. Cells were incubated at 37 °C with 5% CO_2_.

### Cell transfection

siRNAs were transfected using Dharma-FECT 1 Transfection Reagent (GE Dharmacon, T-2001-03) following the manufacturer’s guidelines. The shRNA or COX2 (OriGene Technologies Inc) plasmid was transfected using Lipofectamine 3000 Transfection Reagent (Invitrogen, L3000008) following the manufacturer’s guidelines. The target sequences were listed in **Supplementary Table 1**.

### RT-qPCR

Cells were processed to extract total RNA with the RNeasy Mini Kit (Qiagen, 74104), followed by reverse transcription into cDNA using RT Master Mix (MCE, HY-K 0511). qPCR amplification was conducted using SYBR (MCE, HY-K 0522) on the Applied Biosystems 7500 FAST Real-Time PCR System. The primers sequences were listed in **Supplementary Table 2**.

### Immunohistochemistry

Tissue slides (**Supplementary Table 3**) were used to perform immunohistochemistry staining in accordance with methods described in the literature (13). Inclusion criteria: Pathologically confirmed primary NPC; no prior surgery, radiotherapy or chemotherapy before admission; no history of NPC; complete data. Exclusion criteria: Radiotherapy, chemotherapy or other clinical interventions prior to surgery; severe organ dysfunction; autoimmune diseases, hematological diseases, or history of other malignant tumors; incomplete data. The German semi-quantitative scoring system was used to evaluate staining. Staining intensity was evaluated on a scale from 0 to 3 (0: none, 1: weak, 2: moderate, 3: strong), while the percentage of positive cells was assessed on a scale from 0 to 4 (0: 0%, 1: 1–24%, 2: 25–49%, 3: 50–74%, 4: 75–100%).

### Western blotting

The Western blotting experiment followed the procedures outlined in a prior study (14). Initially, RIPA lysis buffer was used to extract total proteins from the treated cells. The PVDF membrane was incubated with the primary antibody overnight at 4 °C, then with the secondary antibody for 2 hours at room temperature the next day. The membrane was ultimately developed with Pierce™ ECL Western Blotting Substrate.

### Cell viability assay

Transfer the prepared cells into a 96-well plate and incubate them in a cell culture incubator. After culturing, introduce 10 μl of CCK-8 reagent to each well, incubate for 2 hours, and measure the optical density at 450 nm with a microplate reader.

### Colony formation assay

The cells were seeded in a 6-well plate. After culturing for 12–14 days, the cells were fixed with 4% paraformaldehyde and stained with crystal violet. Colonies with more than 50 cells were counted.

### Measurement of lactate levels

The lactate level was measured using the Lactate Assay Kit with WST-8 (Beyotime, S0208S) following the manufacturer’s guidelines.

### Apoptosis analysis

Apoptosis levels in the cells were assessed using the Cell Death Detection ELISA^PLUS^ kit (Roche, 11684795910).In accordance with the kit instructions, positive controls, negative controls and background controls were set up simultaneously. Cell lysates were added to the kit’s microplates and incubated at room temperature with gentle shaking for 2 hours, then unbound immunoreactants were removed. Finally, the samples were analysed using a multi-function microplate reader to calculate the relative apoptosis rate.

### ChIP-qPCR

A ChIP assay kit (Beyotime, P2078) was used following the manufacturer’s guidelines. Cells were fixed with 1% formaldehyde for 10 minutes, followed by a 10-minute treatment with Glycine Solution.Cells were washed with 1 mM PMSF PBS and resuspend in 1 mM PMSF SDS Lysis Buffer, and then lysed using ultrasonic method.Resuspend in ChIP Dilution Buffer and incubate with Protein A+G Agarose/Salmon Sperm DNA for 30 minutes at 4 °C. Then, centrifuged and the supernatant were incubated with 1 μg anti-H3K18la over night at 4°C. The mixture was then incubated with Protein A+G Agarose and Salmon Sperm DNA for 60 minutes at 4 °C. Afterwards, the mixture was centrifuged and removed the supernatant, the precipitate was detected by qPCR.

### RIP-qPCR

BeyoRIP™ RIP Assay Kit (Beyotime, P1805S) was used in accordance with the maufacturer’s guidelines. Briefly, anti-IGF2BP1 or anti-IgG was mixed with Protein A/G Magnetic Agarose Beads overnight at 4 °C. After RNA purification, it was reverse-transcribed into cDNA, target was determined by qPCR.

### mRNA stability assay

The cells were treated with 10 μg/mL actinomycin D for 1, 4, 8, and 12 hours to inhibit transcription. Cells were collected at specified time points and analyzed using RT-qPCR.

### NPC animal model

4-weeks old male BALB/c nude mice were used for NPC animal model (15), which established by subcutaneously injecting nude mice with C666–1 cells expressing the shLDHA or negative control. The volume (mm3) of the implanted tumor was calculated as (width2 × length)/2.

### Statistical analysis

GraphPad Prism software version 5.0 was used for statistical analysis. Results are presented as mean ± standard error of the mean (SEM) from a minimum of three independent experiments. A Student’s t-test was employed for two-group comparisons, while one-way ANOVA was utilized for comparing multiple groups. A P value below 0.05 was deemed statistically significant.

## Result

### The level of H3K18la histone lactylation increases as NPC progresses

**Figure 1:**
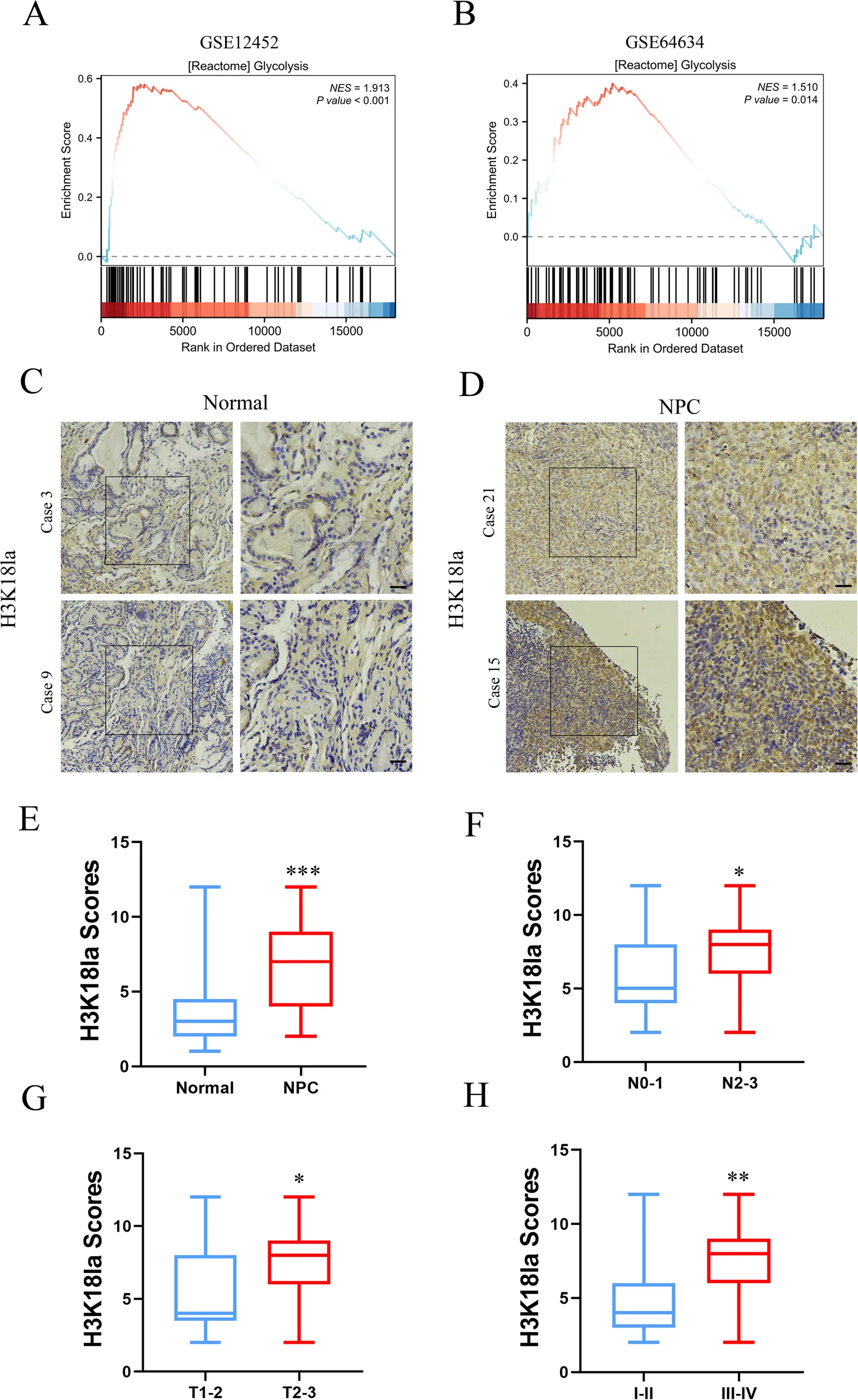
Activation of the glycolytic pathway and high expression of H3K18la in NPC are associated with tumour progression. **(A)** The glycolytic pathway is significantly enriched in dataset GSE12452 **(B)** The glycolytic pathway is significantly enriched in dataset GSE64634 **(C–E)** Representative images and summary data of H3K18la IHC staining in normal (n=30) and NPC tissues (n=36) (scale bar=100µm) **(F–H)** Summary data of H3K18la scores in NPC tissues of different N stages, T stages and clinical stages. *p < 0.05, **p < 0.01, ***p < 0.001, compared to control.

Given that glycolysis is the main pathway for lactate production, which is crucial for protein lactylation, we performed a GSEA on glycolysis-related genes using NPC datasets (GSE12452 and GSE64634) from the GEO database to investigate lactylation’s role in NPC. As depicted in **Figures 1A, B**, glycolysis was significantly enriched in both datasets, with NES of 1.913 and 1.5, respectively. The enrichment curves demonstrate that genes associated with glycolysis are predominantly concentrated at the forefront of the ranked list, indicating a coordinated upregulation of glycolysis and its regulatory proteins in NPC. Although the NES and significance level of dataset GSE64634 were lower compared to those of GSE12452, both independent datasets consistently exhibited positive enrichment patterns, reinforcing the credibility of glycolysis activation in NPC. Furthermore, we employed immunohistochemistry to assess the levels of H3K18la in clinical samples of NPC. The study demonstrated significantly higher H3K18la expression in NPC tissues relative to normal tissues (**Figures 1C–E**), and EBV status may not affect H3K18la levels in NPC tissues (**Supplementary Figure 1**). Further analysis, incorporating clinical and pathological parameters, indicated a strong correlation between H3K18la expression levels and tumor progression. Patients with advanced N-stage, T-stage, and clinical stage displayed significantly higher H3K18la levels than those in the early stages (**Figures 1F–H**). These results suggest that H3K18la acetylation levels in NPC progressively increase in conjunction with tumor progression.

### Lactate promotes H3K18la modification and enhances NPC cells growth

**Figure 2:**
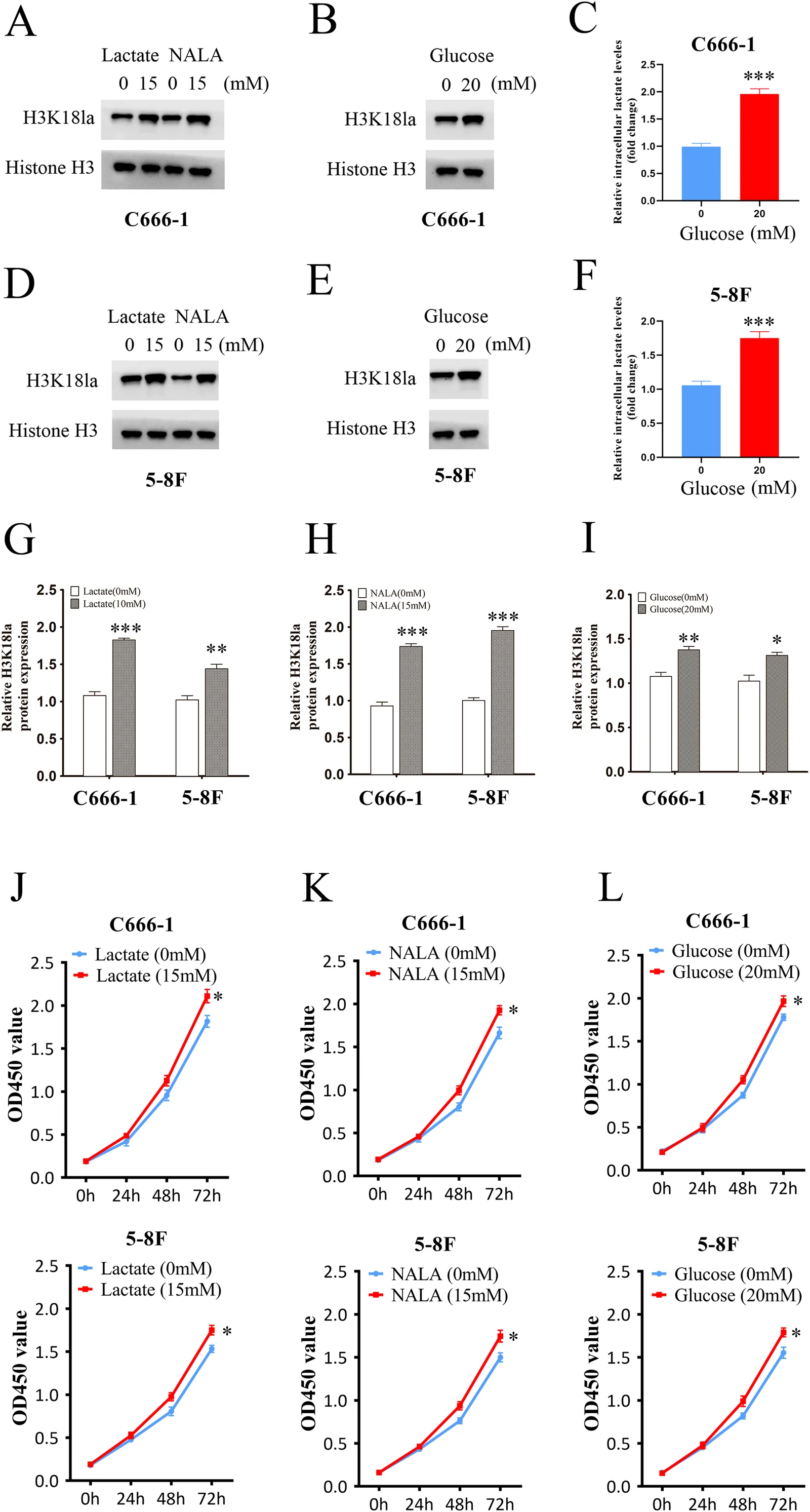
Lactate promotes H3K18la modification and enhances the cell viability of NPC cells**. (A, B, G–I)** Western blot analysis of H3K18la changes in C666–1 cells following treatment with lactate, NALA and glucose for 24h (n=3). **(C)** Measurement of intracellular lactate concentration in C666–1 cells following glucose treatment for 24h (n=4). **(D, E, G–I)** Western blot analysis of H3K18la changes in 5-8F cells following lactate, NALA and glucose for 24h (n=3). **(F)** Measurement of intracellular lactate concentration in 5-8F cells following glucose treatment for 24h (n=4). **(J–L)** CCK-8 assay to assess the proliferation capacity of C666–1 and 5-8F cells following treatment with lactate, NALA and glucose (n=4). *p < 0.05, **p < 0.01, ***p < 0.001, compared to control.

We investigated the role of H3K18la in NPC malignancy using C666–1 and 5-8F cell lines *in vitro*. Cells were initially treated with lactate, NALA, and glucose, and changes in H3K18la modification levels were evaluated using Western blot analysis. The findings revealed that, in comparison to the control group, treatment with lactate, NALA, and glucose resulted in a significant upregulation of H3K18la expression levels in NPC cells (**Figures 2A–E, G–I**). Subsequently, having established that glucose induces the upregulation of H3K18la, we quantified intracellular lactate concentrations. The data showed that glucose significantly increased lactate levels in both C666–1 and 5-8F cells (**Figures 2C, F**). The findings indicate that elevating intracellular lactate, via direct supplementation or enhanced glycolysis, effectively increases H3K18la modification in NPC cells. We assessed the viability of NPC cells using the CCK-8 assay to clarify the effects of histone lactylation on their biological function. The results demonstrated that treatment with lactate, NALA, and glucose significantly increased the viability of NPC cells, suggesting that lactate-induced H3K18la modification may enhance the NPC cells growth (**Figures 2J–L**).

### LDHA inhibition suppresses NPC cell growth by downregulating H3K18la

**Figure 3:**
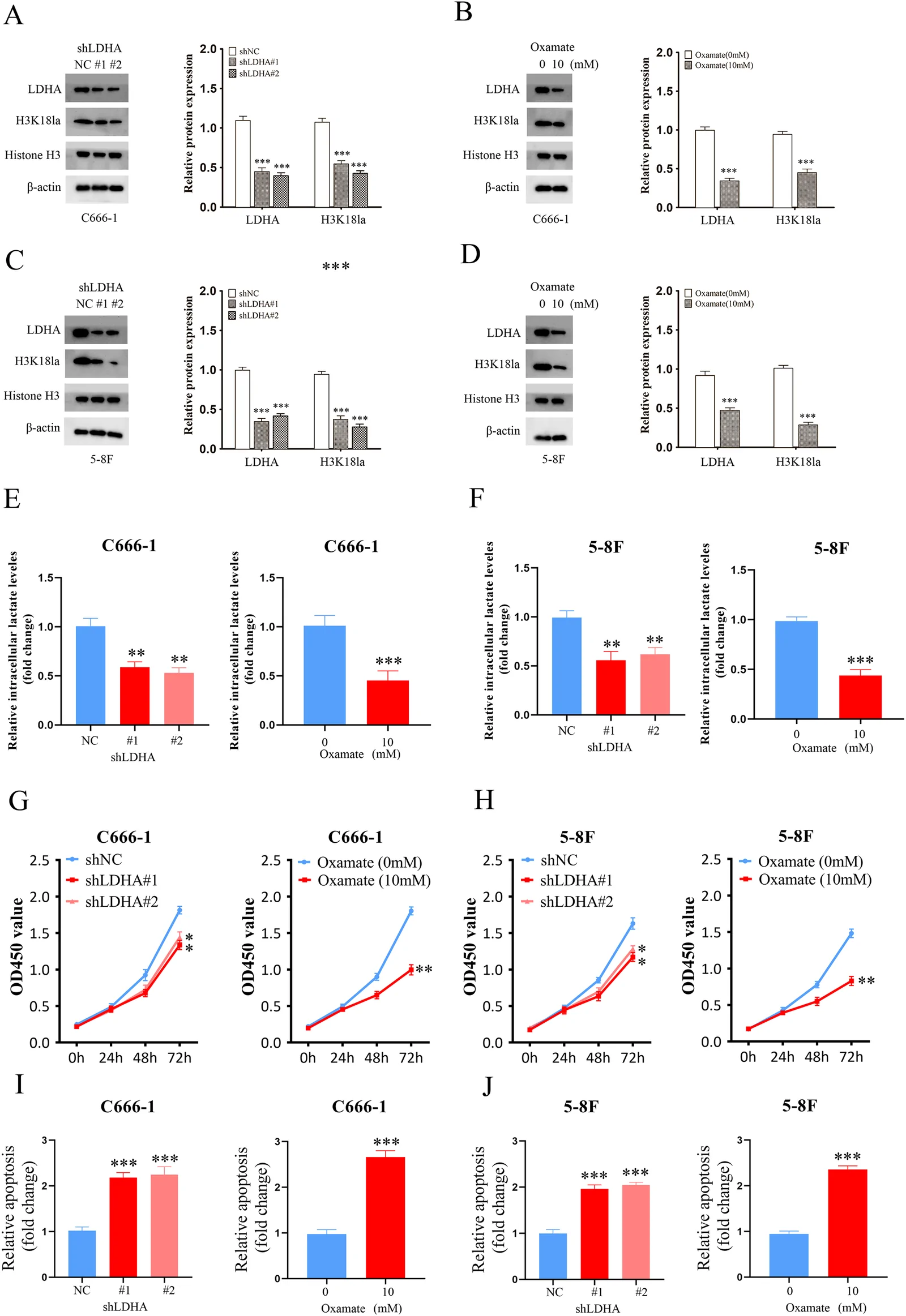
LDHA inhibition downregulates H3K18la and inhibits NPC cell proliferation and induces apoptosis. **(A, B)** Western blot analysis of LDHA and H3K18la levels and intracellular lactate concentration in C666–1 cells with LDHA shRNA (n=3). **(C, D)** Western blot analysis of LDHA and H3K18la levels and intracellular lactate concentration in C666–1 cells with Oxamate for 24h (n=3). **(E, F)** Western blot analysis of LDHA and H3K18la levels and intracellular lactate concentration in 5-8F cells with LDHA shRNA (n=3). **(G, H)** Western blot analysis of LDHA and H3K18la levels and intracellular lactate concentration in 5-8F cells with Oxamate for 24h (n=3). **(I, J)** CCK-8 assay assessing proliferation capacity in C666–1 or 5-8F cells following LDHA shRNA or Oxamate treatment for 24h (n=4). **(K, L)** Assessment of apoptosis levels in C666–1 or 5-8F cells following LDHA shRNA or Oxamate treatment for 24h (n=4). *p < 0.05, **p < 0.01, ***p < 0.001, compared to control.

**Figure 4:**
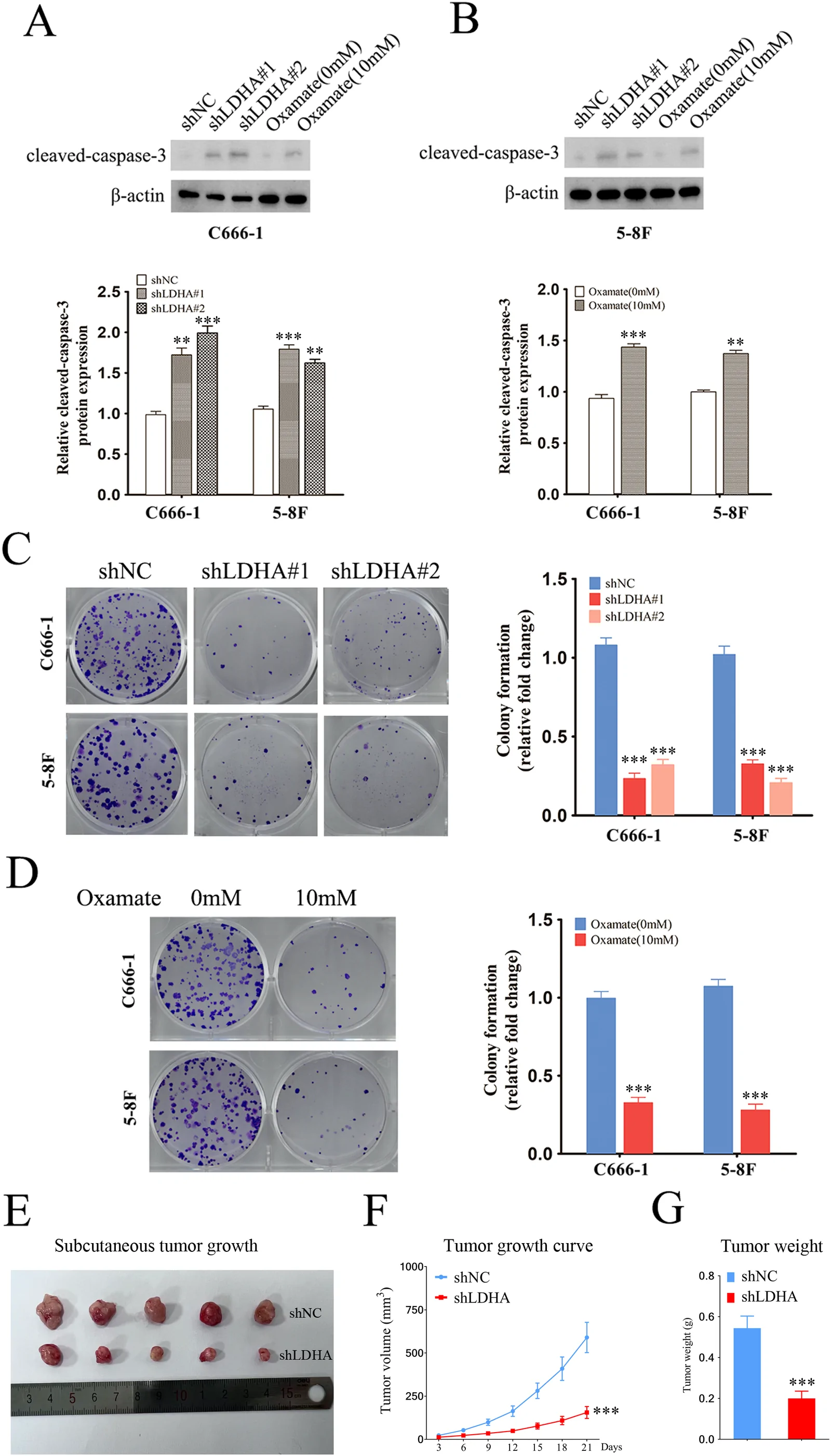
LDHA inhibition suppresses NPC cell growth *in vitro* and *in vivo.* **(A, B)** Western blot analysis of cleaved-caspase-3 levels in C666–1 cells with LDHA shRNA or Oxamate for 24h (n=3). **(C, D)** Colony formation assay in C666–1 cells with LDHA shRNA or Oxamate (n=3). **(E–G)** Tumor image, tumor trace, and tumor weight in the indicated groups (n=5). **p < 0.01, ***p < 0.001, compared to control.

To determine whether LDHA-mediated lactate production regulates the malignant phenotype of NPC via H3K18la modification, we first used shRNA to knockdown LDHA expression in C666–1 cells. Western blot analysis indicated a significant reduction in H3K18la modification levels after LDHA knockdown compared to the negative control (NC) (**Figure 3A**). This confirms that LDHA exerts a specific regulatory effect on histone lactylation. To further validate these findings, we treated the cells with the LDHA inhibitor Oxamate. The results showed that following Oxamate treatment decreased H3K18la levels, consistent with the shRNA results (**Figure 3B**). We also found inhibition of LDHA decreased H3K18la levels in 5-8F cells (**Figures 3C, D**). Furthermore, following LDHA knockdown or inhibition, relative intracellular lactate levels also decreased (**Figures 3E, F**). In functional assays, LDHA knockdown significantly inhibited the cell viability of NPC cells. Similarly, following treatment with Oxamate, the cell viability of the cells also showed a significant decline (**Figures 3G, H**). Apoptosis assays demonstrated that both LDHA knockdown and Oxamate treatment significantly increased apoptosis in NPC cells compared to the control group (**Figures 3I, J**). Moreover, cleaved-caspase-3 expression was increased in NPC cells with shLDHA or Oxamate (**Figures 4A, B**). We also found the LDHA suppression decrease the number of colonies formed in C666–1 and 5-8F cells (**Figures 4C, D**). In line with our *in vitro* findings, LDHA knockdown inhibited NPC tumor growth in an *in vivo* animal model, as shown by decreases in tumor volume and weight (**Figures 4E–G**). These results indicate that LDHA inhibition reduces the cell growth and induces apoptosis by reducing intracellular lactate levels and H3K18la modification in NPC cells.

### H3K18la promotes malignant progression in NPC by enhancing IGF2BP1 expression

**Figure 5:**
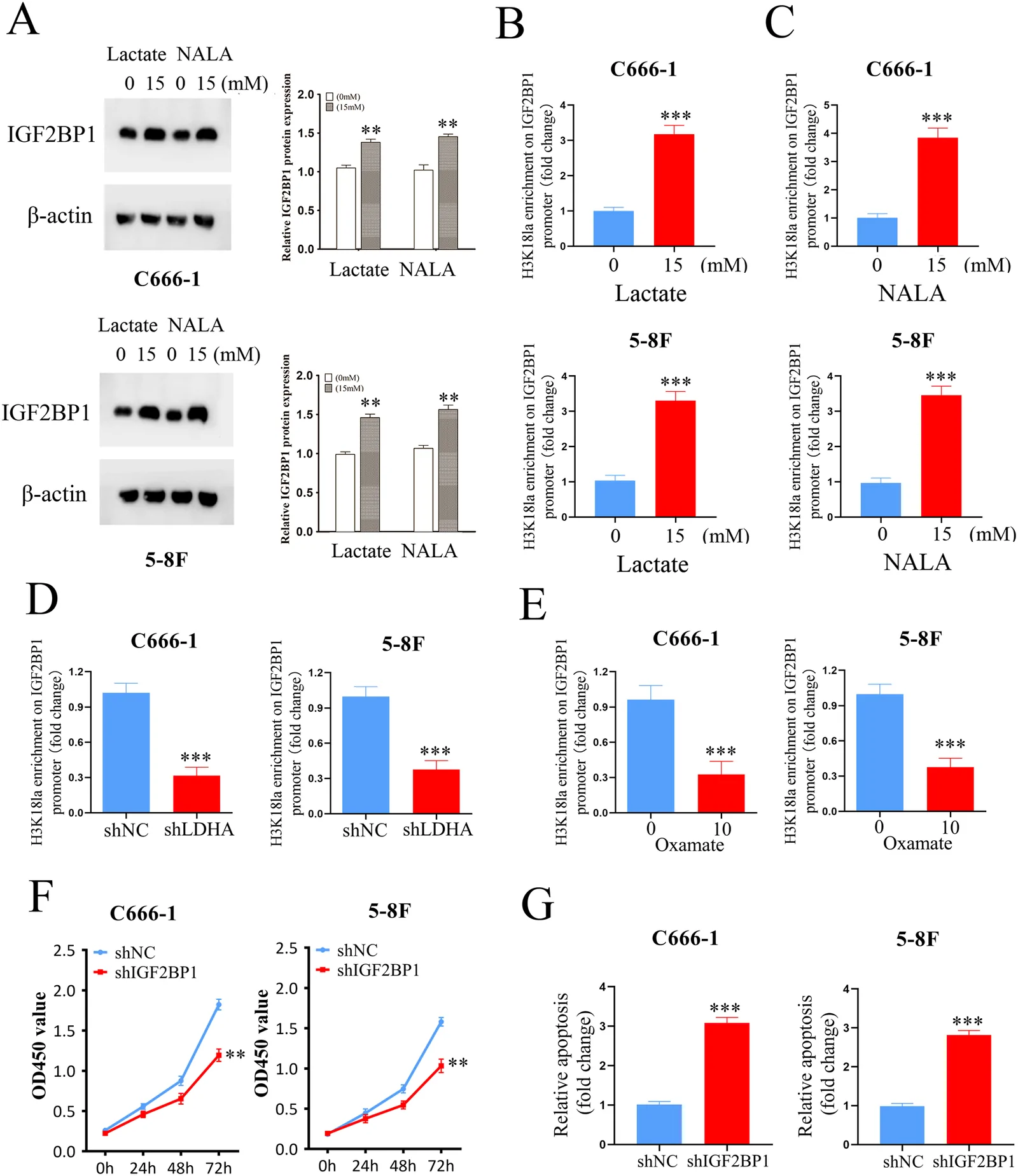
H3K18la promotes malignant progression of NPC by activating IGF2BP1 expression. **(A)** Western blot analysis of IGF2BP1 protein levels in C666–1 and 5-8F cells with lactate and NALA for 24h (n=3). **(B, C)** ChIP-qPCR analysis of H3K18la on IGF2BP1 promoter in C666–1 and 5-8F cells with lactate and NALA for 24h (n=3). **(D, E)** ChIP-qPCR analysis of H3K18la on IGF2BP1 promoter in C666–1 and 5-8F following LDHA shRNA or Oxamate treatment for 24h (n=3) **(F)** CCK-8 assay assessing the effect of IGF2BP1 knockdown in C666–1 and 5-8F cells **(G)** The effect of IGF2BP1 knockdown on the apoptosis levels in C666–1 and 5-8F cells (n=4). **p < 0.01, ***p < 0.001, compared to control.

To elucidate the downstream molecular mechanisms of H3K18la in NPC, we performed Western blot analyses on NPC cell lines treated with lactate and NALA. The study demonstrated that lactate or NALA treatment increased IGF2BP1 protein expression in C666–1 and 5-8F cells (**Figure 5A**). We employed ChIP-qPCR to examine H3K18la enrichment in the IGF2BP1 promoter region, aiming to determine if this regulation is transcriptionally mediated. The findings indicated that lactate and NALA treatment markedly enhanced H3K18la enrichment in the IGF2BP1 promoter region (**Figures 5B, C**), and control gene was not affected (**Supplementary Figure 2**). To confirm these findings, ChIP-qPCR was conducted on NPC cells after LDHA knockdown and Oxamate treatment. The findings indicated a significant reduction in H3K18la enrichment at the IGF2BP1 promoter region in both the shLDHA and Oxamate groups compared to the control group (**Figures 5D, E**). Additionally, lactate increased IGF2BP1 expression in LDHA knockdown NPC cells (**Supplementary Figure 3**). Knockdown of IGF2BP1 expression notably reduced cell viability and triggered apoptosis in NPC cells (**Figures 5F, G**). These results indicate that lactate may regulate NPC cell growth through H3K18la-IGF2BP1 axis.

### IGF2BP1 regulates COX2 mRNA stability to control NPC cells growth

**Figure 6:**
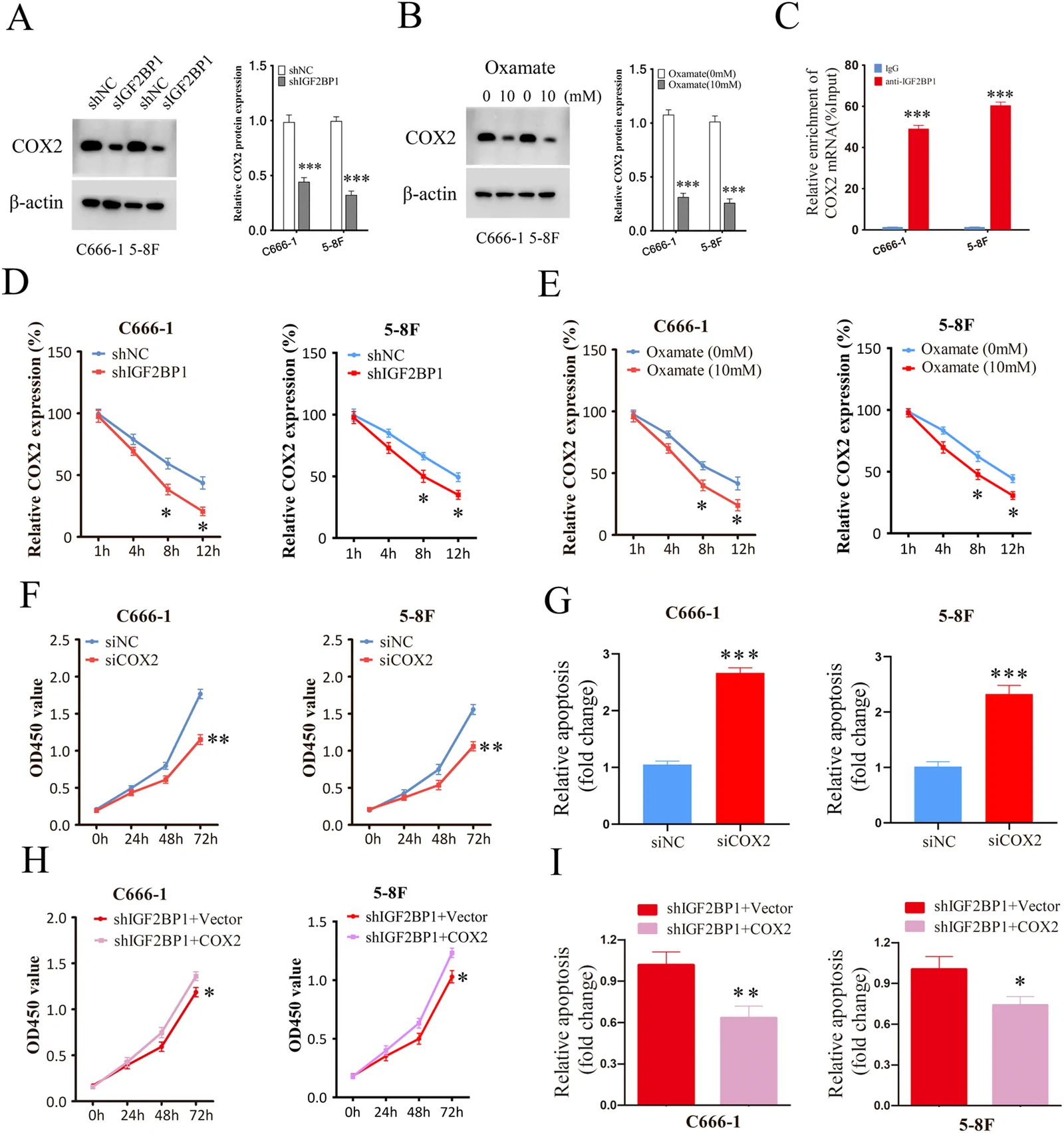
IGF2BP1 influences NPC cell progression by stabilizing COX2 mRNA. **(A)** Changes in COX2 protein levels following IGF2BP1 knockdown in C666-1 (and 5-8F cells (n=3). **(B)** Changes in COX2 protein levels following Oxamate treatment for 24h in C666–1 and 5-8F cells (n=3). **(C)** RIP-qPCR quantification of IGF2BP1-COX2 interaction in C666–1 and 5-8F cells (n=3). **(D)** Changes in mRNA stability of COX2 following IGF2BP1 knockdown in C666–1 and 5-8F cells (n=4). **(E)** Changes in mRNA stability of COX2 in C666–1 and 5-8F cells following Oxamate treatment for 24h (n=4). **(F)** CCK-8 assay to assess the effect of COX2 knockdown on the proliferation capacity of C666–1 and 5-8F cells (n=4). **(G)** The effect of COX2 knockdown on the apoptosis levels of C666–1 and 5-8F cells (n=4). **(H)** CCK-8 assay to assess the effect of COX2 overexpression on the proliferation capacity of C666–1 and 5-8F cells with shIGF2BP1 (n=3). **(I)** The effect of COX2 overexpression on the apoptosis levels of C666–1 and 5-8F cells with shIGF2BP1 (n=3). *p < 0.05, **p < 0.01, ***p < 0.001, compared to control.

To elucidate the downstream mechanisms of the H3K18la-IGF2BP1 axis, we further validated potential downstream target, COX2. Western blot analysis revealed a significant reduction in COX2 protein expression levels in C666–1 and 5-8F cells following IGF2BP1 knockdown, compared to the control group (**Figure 6A**). Oxamate also significantly downregulated COX2 expression (**Figure 6B**). Furthermore, binding IGF2BP1 to COX2 mRNA was measured by RIP-PCR in NPC cells. The result confirmed that IGF2BP1 can bind to COX2 mRNA (**Figure 6C**). Subsequently, we assessed COX2 mRNA degradation rates with IGF2BP1 knockdown using mRNA stability assay (**Figure 6D**). The results indicate that IGF2BP1 knockdown suppressed COX2 mRNA stability. Similar results were shown in Oxamate treatment groups (**Figure 6E**). To further elucidate the functional role of COX2 within this axis, we knocked down COX2 expression using siRNA. CCK-8 assay results showed that, compared with siNC, the cell viability was significantly reduced following COX2 knockdown (**Figure 6F**). Concurrently, COX2 knockdown increased apoptosis levels in NPC cells (**Figure 6G**). Moreover, COX2 overexpression could attenuate the effect of shIGF2BP1 in NPC cells (**Figures 6H, I**). These findings suggested that COX2 may be a key downstream molecule of H3K18la-IGF2BP1 axis, which regulates NPC cells growth.

## Discussion

NPC is a malignant tumor arising from the nasopharynx’s mucosal epithelium, noted for its unique geographical and racial prevalence (2). The World Health Organization classifies NPC into three subtypes: keratinizing, non-keratinizing, and basal cell carcinoma (16). The primary challenges in treating NPC are malignant progression, which often result in treatment failure (17). Additionally, due to the tumor’s specific location, radiotherapy and chemotherapy are frequently associated with significant acute and chronic toxicities. These adverse effects include skin fibrosis, hearing impairment, oral mucositis, and xerostomia, all of which severely affect patients’ long-term quality of life (18). A thorough exploration of the molecular mechanisms behind NPC’s malignant progression and the creation of precision treatments based on molecular subtyping are crucial for enhancing patient outcomes and addressing existing treatment challenges. This study comprehensively explored the expression patterns, clinical relevance, and molecular mechanisms of the H3K18la-IGF2BP1-COX2 signaling axis in NPC cells.

Recent research has identified tumor metabolic reprogramming as a critical feature driving malignant tumor progression (19). In NPC, tumor cells prominently display the “Warburg effect, “ favoring glycolysis for energy production despite sufficient oxygen, resulting in substantial lactate accumulation in the tumor microenvironment (20, 21). Lactate has traditionally been viewed as the final product of tumor glycolysis, contributing to tumor progression by acidifying the microenvironment. However, a study by Zhang et al. Lactate acts as a substrate for histone post-translational modification, influencing gene transcription via lactylation, thus providing new insights into the relationship between metabolism and epigenetics (4). Histone lactylation plays a crucial role in the development of ocular melanoma, cervical cancer, colorectal cancer (22–24). Despite the presence of active glycolysis in NPC leading to increased lactate levels, there is a paucity of comprehensive research investigating whether lactate contributes to tumor progression via histone lactylation. We confirmed significant activation of the glycolytic pathway in NPC by Gene Set Enrichment Analysis, and analysis of clinical samples revealed that H3K18la expression levels rise with tumor progression, indicating its potential as a biomarker for NPC malignancy assessment. Notably, this association is not unique to NPC, elevated H3K18la levels have been noted in non-small cell lung cancer and are linked to poor prognosis (8); patients with stable or progressive disease treated with bevacizumab exhibited significantly elevated levels of H3K18la (25); and Li et al. reported a positive correlation between elevated H3K18la levels and advanced stages of pancreatic ductal adenocarcinoma (5). These cross-cancer concordances suggest that H3K18la may serve as a broad-spectrum epigenetic marker of malignant progression. Furthermore, our findings also indicate that lactate enhances H3K18la levels and promotes the growth of NPC cells. The modification of H3K18la through lactylation could be crucial in NPC progression.

LDHA, as a rate-limiting enzyme in glycolysis, plays a pivotal role in lactate production and is crucial for both physiological functions and tumorigenesis (26, 27). Building upon this, we investigated whether LDHA-regulated lactate production influences H3K18la modification. Our study demonstrated that LDHA-mediated lactate production is essential for maintaining H3K18la levels, inhibition of LDHA not only decreased intracellular lactate levels but also downregulated H3K18la modification, thereby reducing NPC cell viability, inducing apoptosis and xenograft tumor growth. These findings form an intriguing parallel with the observations by Wang et al. in periodontal ligament stem cells (28), while Wang et al. demonstrated that H3K18la enhances osteogenic differentiation in a physiological process, our study reveals that the same modification in NPC drove malignant progression in a pathological process. This phenomenon strongly suggests that the functional output of lactylation is highly dependent on cell lineage and microenvironmental context. We demonstrated that lactate facilitates the enrichment of H3K18la at the promoter region of IGF2BP1, while inhibition of LDHA reduces this enrichment. More importantly, reducing IGF2BP1 expression notably decreased cell viability and triggered apoptosis in NPC cells. Consist with our results, IGF2BP1 is significantly expressed in breast cancer and plays a role in regulating tumor angiogenesis (29). IGF2BP1 is reported to function as an RNA-binding protein, stabilizing mRNAs (29). We demonstrated that IGF2BP1 can bind to COX2 mRNA by RIP-qPCR. IGF2BP1 knockdown markedly reduced COX2 expression. Notably, either IGF2BP1 knockdown or inhibition of lactate production attenuated the COX2 mRNA. COX2 knockdown resulted in decreased cell viability and increased apoptosis in NPC cells. Moreover, COX2 overexpression reversed anti-tumor effect of IGF2BP1 knockdown. While these findings are consistent with previous studies that COX2 is overexpressed in NPC, contributing to tumor growth, apoptosis resistance, and unfavorable prognosis (30, 31). Our study lies in the elucidation of the upstream epigenetic mechanism driving COX2 dysregulation. This mechanistic repositioning elevates COX2 from a functional execution node of the metabolism-epigenetics regulatory axis, providing a new rationale for targeting of COX2.

While our study has several limitations, first, our functional experiments were primarily conducted, which are lacking to validate the dynamic regulation of the H3K18la-IGF2BP1-COX2 axis within the tumor microenvironment. Second, we focused on H3K18la as a specific histone lactylation site; however, the potential cooperative contributions of other histone sites or non-histone lactylation targets remain to be systematically explored. Future studies should integrate spatial metabolomics and single-cell epigenomics to map the spatiotemporal dynamics of the lactylation landscape within the NPC microenvironment. Third, we propose that the activation status of the H3K18la-IGF2BP1-COX2 axis may serve as a predictive metabolic biomarker for NPC patients, which will be further validated in the multi-center with enough sample size.

## Conclusion

In summary, this study indicated that LDHA-mediated H3K18la histone lactylation promotes the malignant progression of NPC by activating the IGF2BP1-COX2 signaling axis. Furthermore, targeting mediators of lactylation or blocking the IGF2BP1-COX2 signaling axis may provide new insights into therapy of NPC.

## Data Availability

The original contributions presented in the study are included in the article/**Supplementary Material**. Further inquiries can be directed to the corresponding author.
