# Supplementary material for: H3K18la facilitates nasopharyngeal carcinoma progression through the activation of the IGF2BP1-COX2 signaling pathway
Source: Front Immunol. 2026 Sep 8;17:1864228. doi: 10.3389/fimmu.2026.1864228 (PMC13593861; doi:10.3389/fimmu.2026.1864228)
Supplement: Supplementary file 1 [file SupplementaryFile1.doc]

**Supplementary information**

Supplementary Figure 1. Summary data of H3K18la scores in EBV-negative (n=17) and EBV-positive (n=19) NPC tissues.

Supplementary Figure 2. (A-B) ChIP-qPCR analysis of H3K18la on β-actin promoter in C666-1 and 5-8F cells with lactate for 24h (n=3).

Supplementary Figure 3. (A-B) Western blot analysis of IGF2BP1 protein levels in LDHA knockdown C666-1 and 5-8F cells with lactate for 24h (n=3). **, p<0.01 compared to control.

Table S1 Target sequences used for silencing

| Gene Name | Target sequence |
| --- | --- |
| *LDHA* | shRNA#1: 5’-CCACCATGATTAAGGGTCTTT-3’  shRNA#2 :5’-CCAAAGATTGTCTCTGGCAAA-3’ |
| *IGF2BP1* | siRNA:5’- GGCCCAUAAUAACUUUGUATT-3’ |
| *COX2* | siRNA: 5’-GGUCUGGUGCCUGGUCUGAUGAUGU-3’ |

.

Table S2 Primer sequences used for qPCR

| Gene Name | Primer | Sequence |
| --- | --- | --- |
| *IGF2BP1* | Forward  Reverse | CTTTGTAGGGCGTCTCATTGGC  CCTTCACAGTGATGGTCCTCTC |
| *COX2* | Forward  Reverse | CGGTGAAACTCTGGCTAGACAG  GCAAACCGTAGATGCTCAGGGA |

Table S3 Clinicpathological characteristics of NPC patients

|  | All Patients  (n=36) | |
| --- | --- | --- |
| Characteristic | n | % |
| Age(years) |  |  |
| ≤50 | 9 | 25 |
| >50 | 27 | 75 |
| Sex |  | |
| Male | 25 | 69.4 |
| Female | 11 | 30.6 |
| T stage |  |  |
| T1-T2 | 15 | 41.7 |
| T3-T4 | 21 | 58.3 |
| N stage |  |  |
| N0-1 | 16 | 44.4 |
| N2-3 | 20 | 55.6 |
| TNM stage |  |  |
| I-II | 10 | 27.8 |
| III-IV | 26 | 62.2 |
| EBV |  |  |
| Negative | 17 | 47.2 |
| Positive | 19 | 52.8 |
